# Similarity Index for the Fat Fraction between Breast Milk and Infant Formulas

**DOI:** 10.1021/acs.jafc.1c08029

**Published:** 2022-05-11

**Authors:** Sanna Hokkanen, Alexander D. Frey, Baoru Yang, Kaisa M. Linderborg

**Affiliations:** † Molecular Biotechnology, Department of Bioproducts and Biosystems, School of Chemical Engineering, Aalto University, 02150 Espoo, Finland; ‡ Food Chemistry and Food Development, Department of Life Technologies, University of Turku, 20500 Turku, Finland

**Keywords:** similarity index, bovine milk fat, human milk fat, infant formula, fatty acid composition, phospholipid composition, regioisomerism, sterol composition

## Abstract

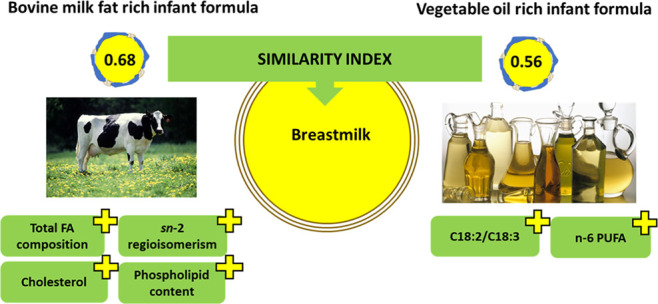

The similarity of the fat fraction in infant formulas rich in either bovine milk fat (MF) or vegetable oil (VO) to breast milk was evaluated by analyzing their lipid composition. Milk fat-rich formulas were highly similar (average similarity index 0.68) to breast milk compared to the VO-rich formulas (average similarity index 0.56). The highest difference in the indices was found in the contents of cholesterol (0.66 vs 0.28 in MF- and VO-rich formulas, respectively, on average) and polar lipids (0.84 vs 0.53), the positional distribution of fatty acids in the *sn*-2 position of triacylglycerols (0.53 vs 0.28), and fatty acid composition (0.72 vs 0.54). The VO-based formulas were superior in similarity in *n* – 6 PUFA. Thus, the addition of bovine MF fractions is an effective way to increase the similarity between the lipid composition of infant formulas and human milk.

## Introduction

Breast milk is the optimal nutrition for the newborn baby providing comprehensively the energy and nutrients needed by the infant. However, breastfeeding is not always possible and infant formulas need to be used. In Europe, the European Food Safety Authority (EFSA) has given an opinion on the compositional requirements of the infant formulas to support the health and development of the infant,^1^ and the composition of the formula is governed by Regulation (EU) No 609/2013. It is possible to obtain the required composition by using a variety of different ingredients, but it is difficult to approximate which ingredient mixture produces the highest similarity to breast milk.

Infants receive roughly 50% of energy from fat in milk or formula.^2^ The fat of human milk is a very complex mixture consisting of at least hundreds of different lipids, and the lipid composition is tailored for optimal adsorption and nutritional value. Saturated fatty acids (FAs) (most abundantly C16:0) are incorporated in the *sn*-2 position of the triacylglycerol (TAG) molecule, which makes their adsorption efficient.^3^ Besides energy, the fat fraction has an important role in brain and eye development, gut health, and immune function. Long chain polyunsaturated FA (LCPUFA) of milk, especially arachidonic acid (ARA) and docosahexaenoic acid (DHA) contribute to the membrane fluidity in the developing brain and have an impact on enzyme activities and receptor function.^4,5^ The phospholipids of the milk fat globule membrane (MFGM) have been shown to improve cognitive performance in infants and provide protection against pathogenic bacteria and their toxins by enhancing the immunity of the gut epithelial cells.^6−8^ Cholesterol, which is also a membrane lipid, has been shown to have positive effects on the lipid metabolism of the infant.^9−11^

Fat in infant formulas is usually a mixture of fats from different sources to fulfill the nutritional recommendations. Originally meant as nutrition for the calf, dairy fat has several components similar to those in breast milk, for example, those related to MFGM. However, dairy fat is not suitable as the sole fat ingredient in the infant formulas due to lower amounts of linoleic acid (LA) and α-linolenic acid (ALA) than required,^1^ and therefore supplementation with vegetable oils (VO) is necessary. In VOs the compositions of phospholipids and sterols are different from milk fats (MFs) and, for example, cholesterol is absent.^12^ Structured fats in which the palmitic acid is enriched in the *sn*-2 position are commonly used to improve the TAG structure of VO-based formulas. Independent of the major fat source used in the infant formulas, ARA and DHA from different origins, for example, from fish oils or single cell oils, are supplemented to formulas according to the regulations on the absolute content of polyunsaturated long chain FAs in infant formula.

While human milk is instantly consumed, formulas are heat-treated to guarantee safety and homogenized with high pressure to maintain emulsion consistency during the shelf-life. Thus, besides differences in the nutritional content, technological reasons make the fat fraction in infant formulas structurally different from that in human milk. In the homogenization of milk, the size of the lipid droplets is reduced and the natural MFGM is partially disrupted. To cover the increased surface area, the dairy proteins, primarily caseins, are adsorbed onto the droplet interphase.^13^ In liquid infant formulas the lipid droplets are under 1 μm in diameter^14^ while in mature human milk, the size range of the fat globules is 0.4–13 μm having an average diameter of 4–5 μm.^14,15^ Even if the larger surface area of the small fat globules offers more substrate to the lipolytic enzymes, there are indications of impaired digestibility of the lipid droplets which have undergone homogenization and thermal treatments.^16^

The similarity index for infant formulas and breast milk was introduced by Al-Abdi et al.^17^ as a tool to evaluate different formulas in respect of the claimed composition, and thus only the content of total fat and certain *n* – 3 and *n* – 6 LCPUFA from the fat fraction were included in the index. Kloek et al.^18^ proposed an extended index taking into account the positional distribution of FA in TAG and also the overall FA composition. However, until now, the indices for the important minor lipid components have remained unevaluated. In this study, we calculated the similarity index for the fat composition including the membrane lipid components (phospholipids and sterols) and size of the fat globules of representative selected infant formulas on the Finnish market and the breast milk of Finnish donors. The formulas and breast milk were analyzed in parallel, which enables a direct comparison of the values.

## Materials and Methods

### Breast Milk and the Infant Formulas

The infant formulas were purchased from a local retail market in Espoo, Finland. All the formulas were intended for infants under 6 months. The formulas were selected on the basis of the fat source indicated in the list of ingredients: three dairy fat (MF)-containing products and three VO-based products. The fats and oils used as ingredients in the formulas are presented in Table 1.

**Table 1:** Infant Formulas Used in the Study^a^

#	form	fat source	emulsifier
MF1	liquid	bovine cream, sunflower oil, milkfat rich whey protein, rapeseed oil, coconut oil, fish oil, *M. alpina* -oil	soy lecithin, mono- and di-glycerides
MF2	liquid	bovine cream, sunflower oil, soy oil, DHA from microalgae	soy lecithin
MF3	liquid	bovine milk, rapeseed oil, sunflower oil, fish oil	sunflower lecithin, mono- and di-glycerides
VO1	liquid	sunflower oil, coconut oil, rapeseed oil, fish oil, *M. alpina* -oil	soy lecithin
VO2	liquid	sunflower oil, coconut oil, rapeseed oil, fish oil, *M. alpina* -oil	not specified
VO3	powder	palm oil, coconut oil, rapeseed oil, sunflower oil, oleic acid-rich sunflower oil, fish oil, *M. alpina* -oil	soy lecithin

a MF, milk-fat-containing formula; VO, the formula containing vegetable oils as the primary fat source.

The study was conducted according to the WMA Declaration of Helsinki. Breast milk of Finnish origin was obtained from volunteer mothers (*n* = 8) living in the Turku area in Finland. Healthy mothers who breastfed an infant younger than 6 months of age were recruited. Only mothers who had given birth to a healthy full-term infant, whose infant had grown normally were accepted. Approval of the study was obtained from the Ethics Committee of the Hospital District of Southwestern Finland (106/1801/2018). All mothers gave informed consent. The milk was collected manually by the mothers from the right breast after milking first drops to waste, after restriction to breastfeed from that breast for 2 h prior to milk collection. Breastfeeding from the left breast was not restricted. Nitrile gloves were used during the self-collection. Milk was either cooled (+6 °C) or frozen (−20 °C) by the mothers and transferred to the research unit, typically during the same day. For all of the analyses excluding the particle size determination, equal amounts of milk from each of the 8 mothers were pooled. For the particle size analysis, due to the sample availability, only one fresh unfrozen milk sample and one frozen milk sample were analyzed.

### Reagents

Silica cartridges (Supelclean LC-Si SPE tube, bed weight 500 mg, volume 3 mL), borontrifluoride (14%)-methanol, pancreatic lipase, sodium cholate, 5β-cholestan-3α-ol (purity min. 95%), Supelco 37 component F.A.M.E. mixture, Sigma 7–9 Tris base, sodium dodecyl sulfate, pyridine, and Rhodamine 6G (95%) were purchased from Sigma-Aldrich, MO, USA; 1,2-dipentadecanoyl phosphatidyl choline, 1-monoheptadecanoin, dipentadecanoin, heptadecanoic acid, triheptadecanoin, tridecanoic acid methyl ester, and sphingomyelin (natural from bovine) were purchased from Larodan, Sweden; dilayryl phosphatidyl ethanolamine, phosphatidyl serine (natural from porcine brain), and phosphatidyl inositol (natural from bovine liver) were purchased from Avantilipids, AL, USA; 20 × 20 cm silica plates (Kieselgel 60), glacial acetic acid, calcium chloride, sodium hydroxide, potassium chloride, and potassium hydroxide were from Merck, Darmstadt, Germany; bis(trimethylsilyl)-trifluoracetamid (BSTFA) and trimethylchlorosilane (TMCS) were purchased from Macherey-Nagel, Dueren, Germany; methyl acetate (99%) was purchased from Acros Organics, Ceel, Belgium; hydrogen chloride (37%), petroleum ether, diethyl ether (>98%), hexane (>99.5%), and heptane (>99%) were purchased from Avantor Performance Materials, Gliwice, Poland; dichloromethane (>99.8%) and methanol (99.9%) were purchased from Honeywell; 1-propanol, 2-propanol, and methyl-*t*-butyl ether (HPLC grade) were purchased from Rathburn Chemicals, Walkerburn, Scotland; sodium sulfate (anhydrous) was from J.T. Baker Chemical Company, Deventer, The Netherlands.

### Fat Extraction

The powdered formula was reconstructed according to the instructions of the package and treated similarly to the liquid formulas. Briefly, 4.6 g of the powder was suspended in 30 mL of distilled water at 40 °C. The suspension was shaken well for 10 s. Lipids from 1 mL of the infant formulas and breast milk were extracted with 4 mL of dichloromethane-methanol (2:1). The suspensions in the capped 10 mL kimax tubes were flushed with nitrogen, vortexed vigorously, and shaken (350 rpm) for 30 min at room temperature. After centrifugation (1500*g*, 5 min) the organic phase was collected in a clean tube, and the aqueous phase was re-extracted with 2 mL of dichloromethane as mentioned above. The organic phase was combined with the organic phase from the first extraction and evaporated to dryness at 30 °C under a nitrogen stream.

### Separation of Neutral and Polar Lipids

Neutral and polar lipids were separated by solid-phase extraction as described previously.^19^ The total lipid extract was dissolved in 0.25 mL of dichloromethane-methanol (2:1) and loaded in the silica cartridge which was conditioned with 4 mL of hexane. The samples intended for the analysis of total phospholipid content were supplemented with 10 μL of the phospholipid standard (1,2-dipentadecanoyl phosphatidyl choline) dissolved in chloroform at a concentration of 10 mg/mL prior to analysis. The neutral lipids were eluted first with 2 mL of hexane-diethylether (4:1) followed by elution with 2 mL of hexane-diethylether (1:1), and the solvent from the combined eluents was evaporated to dryness under a nitrogen stream at 30 °C. The polar lipids were eluted with 2 mL of methanol, followed by 2 mL of dichloromethane/methanol/H_2_O (3:5:2), and evaporated to dryness at 37 °C.

### Separation of Polar Lipid Classes

To ensure the detection of the smallest compounds, duplicate phospholipid samples were combined for TLC separation of the polar lipid classes. The samples were dissolved in 0.1 mL of dichloromethane/methanol (100:1) and applied on the lower edge of the silica plate. The lipids were separated in the chamber containing methyl acetate/dichloromethane/2-propanol/methanol/0.25% KCl (25:25:25:10:9) as elution solvent. After 1 h elution, the plate was let to dry at room temperature and re-eluted for 1 h. The plate was sprayed with aqueous 0.001% rhodamine 6G and the spots containing lipids were visualized under UV light. The lipid spots were recognized by comparing with the elution of the standard lipids (phosphatidyl choline, phosphatidyl ethanolamine, phosphatidyl serine, phosphatidyl inositol, and sphingomyelin; dissolved in chloroform in a concentration of 10 mg/mL), scraped off the plate into the separated tubes, flushed with nitrogen, and stored at −20 °C until further analyzed.

### Content of Total Fat and the Polar Lipid Classes

The total fat content was measured from 0.2 mL of the lyophilized liquid formulas and breast milk or 25 mg of the powdered formula by direct saponification as described previously.^20^ For conversion of FA to methyl esters, total fat and phospholipid classes after separation on TLC were treated similarly. The phospholipid classes were supplemented with 3 μL and total fat samples with 10 μL of C13:0-methyl ester standard, 20 mg/mL. The lipids were saponified with 1 mL of 3.7 M NaOH in 49% methanol by incubating the tubes for 30 min in a boiling water bath. The tubes were flushed with nitrogen prior to incubation. The cooled samples were supplemented with 4 mL of 3.3 M HCl in 48% methanol and flushed with nitrogen. Methylation of the FAs occurred during 30 min incubation at 80 °C. Lipids were extracted to the organic phase by supplementing with 1.5 mL hexane/methyl-*t*-butylether (1:1) and shaken vigorously (350 rpm) at room temperature for 10 min. The organic phase was washed with 10% (w/v) NaOH by shaken for 5 min as mentioned above. For the sharpening the phase boundary, the tubes were centrifuged (1500*g*) for 20 min. The organic phase was dried with anhydrous sodium sulfate and transferred to a GC vial for the determination of methyl ester concentrations. The phospholipids were further concentrated by evaporating the sample in the GC vial to dryness and concentrated in 0.1 mL of hexane.

### Total Phospholipid Content

The polar lipids from solid-phase extraction including the internal standard (dipentadecanoyl-phosphatidyl choline) were methylated with 0.5 mL borontrifluoride (14%)-methanol by incubating the tubes 90 min in a boiling water bath as described previously.^19^ The tubes were flushed with nitrogen prior to incubation. The cooled samples were supplemented with 1 mL deionized H_2_O and 1.5 mL of hexane-methyl-*t*-butylether, and the lipids were extracted to the organic phase as described above. After transfer to the GC vials, the sample was evaporated to dryness and concentrated in 0.1 mL of hexane.

### Separation, Detection, and Calculation of FA Methyl Esters in Lipid Fractions

The methyl esters were separated on a Zebron ZB-FAME column (60 m × 250 μm × 0.2 μm), and an Agilent 7890 A GC system equipped with an FID detector was used as previously described.^19^ The oven temperature was raised gradually to 280 °C. The gas flow in the detector was 350, 30, and 35 mL/min for air, H_2,_ and N_2_, respectively. The split ratio was 10:1. FAs were detected by comparing the elution order to the 37 component F.A.M.E. mix standard. The concentration of the FAs in each lipid class was calculated by comparing the peak areas of the sample methyl esters to the peak area of the methyl ester of the internal standard lipid.

### Regioisomerism of FAs in TAGs

An enzymatic method adapted and modified from Korma et al.^21^ was used in the determination of FAs in the *sn-1/3* and *sn*-2 positions of TAGs. The neutral lipid fraction from solid-phase extraction was heated to 40 °C to liquefy the fats, and 10 mg was weighed in a clean tube by using a glass capillary. By keeping the temperature of the fat sample at all times over 37 °C, the tube was supplemented with 2 mL of preheated (37 °C) lipase-suspension (10 mg/mL of pancreatic lipase 1 M Tris–HCl, pH 8.0), 0.2 mL of 4.4% CaCl_2_, and 0.5 mL of 0.1 mg/mL aqueous sodium cholate. The reaction was carried out at 37 °C in a water bath with magnetic stirring. After 6 min the reaction was stopped by adding 1 mL of 6 N HCl. The lipids were extracted from the hydrolysis suspension with 2 mL of diethyl ether by shaking (350 rpm) for 15 min at room temperature. Prior extraction, 20 μL of the lipid standard mixture (1-monoheptadecanoin 10.4 mg/mL, dipentadecanoin 8.0 mg/mL, heptadecanoic acid 12.0 mg/mL, and triheptadecanoin 11.3 mg/mL), was added. 1-Monoheptadecanoin elutes together with 2-monoacylglycerols and was selected as the standard due to better availability. After centrifugation (1500*g*/5 min) the organic phase was collected in a clean tube and the aqueous phase was re-extracted with 2 mL of diethylether as described above, combined with the extract from the first extraction and evaporated to dryness at 30 °C under a nitrogen stream.

The hydrolyzed lipids were separated on TLC according to Liukkonen et al.^22^ The lipid sample was dissolved in 0.2 mL of dichloromethane/methanol (100:1) and applied on the lower edge of the plate. The lipid classes (monoacylglycerols, diacylglycerols, free FA, and TAG) were separated by using petroleum ether/diethylether/glacial acetic acid (80:30:1) as the elution solvent. Elution time was 1 h. The plate was sprayed with 0.001% aqueous rhodamine 6G and visualized under UV light. The monoacylglycerol and free FA containing lipid spots were scraped off the plate to the separated tubes and flushed with nitrogen. Hydrolysis, saponification, and methylation of the FA in separated lipid classes bound to silica matrix were carried out similarly to the total fat and the polar lipid classes described above. No standard addition was required due to the presence of an internal standard for each lipid class. The concentration of FA in the *sn*-2 and *sn*-1/3 positions was determined by calculating the content of FA (mol %) in monoacylglycerols and free FA, respectively.

### Lipid Droplet Size

Lipid droplet size distribution was measured for liquid infant formulas and breast milk with a Mastersizer 2000 (Malvern Instruments, Malvern, UK). A refractive index of 1.458 was adopted. The analysis was carried out directly upon package opening and after thorough shaking of the products. The lipid droplet size of breast milk was analyzed within 18 h after milking from one milk sample and from one frozen sample including 4 parallel measurements per sample. Three samples per infant formula were analyzed including 4 parallel measurements per sample. The powdered formula was prepared according to the instructions in the package. Of this suspension, 1 mL was diluted with 9 mL of 1% sodium dodecyl sulfate. The light scattering was measured 30 times in 1 min intervals. However, the individual lipid droplets were visible already within the first measurement.

### Sterol Analysis

Sterols in infant formulas and breast milk were analyzed as described by Laakso.^23^ Samples (200 μL) were weighed into kimax-tubes and 10 μL of the internal standard, 5β-cholestan-3α-ol; dissolved in *n*-propanol in concentration 9.97 mg/mL), was added into the sample. The lipids in the sample were saponified by adding 2.5 mL of absolute ethanol and 0.4 mL 22 M KOH. The sealed tubes were incubated for 30 min at 80 °C by vortexing every 2 min. The cooled samples were supplemented with 2 mL of deionized H_2_O and the nonsaponifiable lipid fraction containing the sterols was extracted with 3 mL heptane by vortexing three times 10 s and the tubes were centrifuged for 5 min at 1500*g*. The heptane phase was transferred into a clean tube and the aqueous phase was re-extracted as described above. The heptane was evaporated at 60 °C under nitrogen stream and redissolved in 1 mL of heptane for transfer into a silylated vial. The heptane was evaporated, and the sterols were derivatized with 200 mL BSTFA; containing 1% TMCS by incubation for 15 min at 70 °C. The trimethylsilyl ether derivatives of the sterols were separated on a fused silica capillary column coated with 5% phenyl/95% dimethylpolysiloxane (30 m 6 0.32 mm i.d., film thickness 0.25 mm; HP-5: Agilent Technologies Inc., Little Falls, DE, USA) with GC (Shimadzu GC-2010, Japan). The components were separated isothermally at 300 °C and be detected with the FID (310 °C). The injection volume was 1.0 mL and the split ratio was 1:5. Shimadzu GCsolution software was used for data collection and processing.

### Calculation of Similarity Index

A modified version of Bray–Curtis similarity index introduced by Al-Abdi et al.^17^ was used to calculate the similarity index for fat fraction: ASI(fat) between breast milk and infant formulas. The modified formula, in which the average similarity index (ASI) is calculated, takes into account the heterogenous measure units of the selected elements
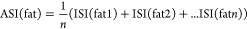
1where ASI(fat) is the ASI and individual similarity indexes (ISI)(fat1...fat*n*) are the individual similarity indices of the selected fat elements. According to Bray and Curtis^24^
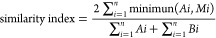
We denote the smaller value as *f* and the higher value as *F*, and thus, each ISI can be presented as
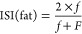
for example, total fat in MF1 is 3.17%, which gives the following value (and that in breast milk is 3.32%)
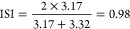


## Results and Discussion

This study evaluated the similarity between six selected infant formulas and a pooled Finnish breast milk sample. Three MF-containing formulas and three VO-based formulas were selected and analyzed for the total content of FA, FA composition, TAG positional regioisomerism, phospholipid content and composition, sterol content and composition, and lipid droplet size. All seven samples were analyzed parallelly, and the similarity index was calculated for each lipid element individually. The ASI for fat fraction was calculated by averaging the ISI.

### Similarity Index for Total Fat Content and Composition

Breast milk is an o/w emulsion containing 3–4% fat packed in the fat globules.^25^ According to the package labels, the studied infant formulas contained 3.4–3.6% fat (Table 2). We measured the total content of FA in the breastmilk and the formulas, and the values for the formulas were 3.0–3.2 g/100 g (Table 2) while the pooled breast milk sample contained 3.3 g/100 g FA, which reflects the total fat content of the milks. In our study, the FA content was determined by direct methylation of the FAs and calculating the masses of the FAs by using an internal standard. This method takes into account only FAs, but, for example, sterols and fat-soluble vitamins are not included, and thus our value cannot be directly compared to the total fat content. It has also been noted that the methylation method may play a role in the determined total quantity of FAs.^26^ However, because all of our samples were analyzed in the same batch, the results are comparable and relevant for similarity index calculations. The similarity index for total FA content, ISI (FA, g/100 g) (Table 3), was high in all of the formulas because the fat content is a simple parameter to adjust in the product development (proposed minimum and maximum contents 2.6 and 4.2% (calculated from the given value per 100 kcal), respectively laid down by Directive 2006/141/EC).

**Table 2:** Total Fat Content, Polar Lipid Content, Lipid Droplet Size, and Fatty Acid Composition of the Infant Formulas and Breast Milk^a^

	MF1	MF2	MF3	VO1	VO2	VO3	breast milk^b^
total FA content (g/100 g)	3.2 ± 0.4	3.1 ± 0.4	3.0 ± 0.2	3.1 ± 0.3	3.2 ± 0.3	3.2 ± 0.1	3.3 ± 0.07
labeled total fat (%)	3.5	3.6	3.5	3.6	3.4	3.4	
polar lipids (mg/g)	0.67 ± 0.03	0.50 ± 0.04	0.60 ± 0.02	0.65 ± 0.06	0.14 ± 0.03	0.18 ± 0.02	0.56 ± 0.04
lipid droplet size (μm, D[4,3])	0.31 ± 0.00	0.44 ± 0.01	0.36 ± 0.00	0.48 ± 0.00	0.44 ± 0.00	2.4 ± 0.1	5.4 ± 0.0^c^
Fatty Acid Composition (%)							
C4:0	0.10 ± 0.10	0.13 ± 0.10	0.16 ± 0.13	0.00 ± 0.00	0.00 ± 0.00	0.00 ± 0.00	0.00 ± 0.00
C6:0	0.19 ± 0.19	0.15 ± 0.15	0.28 ± 0.28	0.04 ± 0.04	0.03 ± 0.03	0.10 ± 0.01	0.02 ± 0.01
C8:0	0.81 ± 0.38	0.36 ± 0.23	0.43 ± 0.24	1.09 ± 0.57	0.75 ± 0.46	1.59 ± 0.03	0.10 ± 0.01
C10:0	1.35 ± 0.25	1.12 ± 0.31	1.30 ± 0.31	1.03 ± 0.36	0.81 ± 0.11	1.38 ± 0.03	1.03 ± 0.01
C12:0	5.63 ± 0.70	1.57 ± 0.20	1.88 ± 0.10	8.36 ± 1.50	7.63 ± 1.01	11.92 ± 0.18	4.27 ± 0.02
C14:0	5.68 ± 0.34	4.97 ± 0.24	6.49 ± 0.38	3.40 ± 0.20	3.21 ± 0.22	5.46 ± 0.09	5.77 ± 0.05
C14:1	0.37 ± 0.03	0.48 ± 0.04	0.66 ± 0.06	0.02 ± 0.01	0.00 ± 0.00	0.02 ± 0.00	0.30 ± 0.00
C15:0	0.38 ± 0.01	0.43 ± 0.01	0.58 ± 0.02	0.06 ± 0.00	0.04 ± 0.00	0.06 ± 0.00	0.36 ± 0.00
C16:0	14.83 ± 0.22	15.33 ± 0.24	21.18 ± 0.28	6.53 ± 0.27	6.15 ± 0.10	19.55 ± 0.05	21.97 ± 0.14
C16:1	0.68 ± 0.03	0.64 ± 0.02	0.97 ± 0.04	0.21 ± 0.00	0.16 ± 0.01	0.17 ± 0.00	2.14 ± 0.02
C17:0	0.20 ± 0.02	0.22 ± 0.01	0.30 ± 0.02	0.06 ± 0.00	0.04 ± 0.00	0.08 ± 0.00	0.27 ± 0.00
C17:1	0.12 ± 0.00	0.13 ± 0.00	0.17 ± 0.02	0.05 ± 0.00	0.04 ± 0.00	0.05 ± 0.00	0.19 ± 0.00
C18:0	6.23 ± 0.86	4.95 ± 0.51	8.12 ± 1.04	2.51 ± 0.33	2.57 ± 0.27	2.59 ± 0.13	4.74 ± 0.04
C18:1	42.06 ± 1.33	45.16 ± 0.46	30.03 ± 0.40	56.07 ± 2.28	58.60 ± 1.13	39.13 ± 0.51	42.74 ± 0.24
C18:2*n*-6	18.29 ± 0.55	21.52 ± 0.42	24.34 ± 0.56	18.13 ± 0.51	17.43 ± 0.55	14.72 ± 0.15	12.33 ± 0.10
C18:3*n*-3	1.88 ± 0.13	1.89 ± 0.12	1.92 ± 0.10	1.52 ± 0.09	1.68 ± 0.15	1.75 ± 0.03	1.82 ± 0.02
C20:0	0.12 ± 0.04	0.14 ± 0.06	0.15 ± 0.07	0.10 ± 0.06	0.10 ± 0.06	0.04 ± 0.00	0.24 ± 0.04
C20:1	0.07 ± 0.06	0.07 ± 0.03	0.10 ± 0.05	0.10 ± 0.10	0.09 ± 0.12	0.21 ± 0.01	0.33 ± 0.06
C20:2	0.03 ± 0.03	0.02 ± 0.02	0.02 ± 0.02	0.00 ± 0.00	0.01 ± 0.01	0.04 ± 0.00	0.24 ± 0.01
C20:4*n*-6	0.25 ± 0.01	0.03 ± 0.01	0.07 ± 0.01	0.03 ± 0.02	0.22 ± 0.01	0.46 ± 0.01	0.31 ± 0.04
C22:5n-3	0.03 ± 0.00	0.03 ± 0.01	0.04 ± 0.01	0.01 ± 0.01	0.00 ± 0.00	0.02 ± 0.00	0.16 ± 0.00
C22:6*n*-3	0.22 ± 0.11	0.35 ± 0.05	0.29 ± 0.03	0.36 ± 0.04	0.15 ± 0.02	0.36 ± 0.00	0.27 ± 0.01
other^d^	0.47 ± 0.06	0.28 ± 0.04	0.53 ± 0.05	0.33 ± 0.05	0.28 ± 0.04	0.29 ± 0.01	0.40 ± 0.01
C16:0 in *sn*-2 position (mol % of total C16:0)	26.9 ± 0.3	27.9 ± 0.5	34.5 ± 0.4	7.9 ± 0.2	6.3 ± 0.8	11.3 ± 0.1	72.6 ± 1.4
SCSFA + MCSFA^e^	8.07	3.35	4.05	10.52	9.22	15.00	5.41
LCSFA^f^	27.4	26.0	36.8	12.7	12.1	27.8	33.4
total SFA	35.5	29.4	40.9	23.2	21.3	42.8	38.8
total MUFA	43.3	46.5	31.9	56.4	58.9	39.6	45.7
*n* – 6 PUFA	18.5	21.6	24.4	18.2	17.7	15.2	12.6
*n* – 3 PUFA	2.1	2.3	2.2	1.9	1.8	2.1	2.2
LA/ALA	9.8	11.5	12.7	12.0	10.4	8.4	5.5
							

a Values are average (*n* = 4) ± SD; MF, milk-fat-containing formula; VO, the formula containing vegetable oils as the primary fat source.

b Pooled sample of mothers’ milk from eight donors from Finland.

c One fresh and one frozen breast milk sample.

d C11:0, C15:1, C18:3*n*-6, C21:0, C20:3, C22:0, C22:1, C20:5, C20:3, C23:0, C22:2, C22:4*n*-6, C24:0, and C24:1).

e Short chain and medium chain FA (C4:0, C6:0, C8:0, C10:0, and C12:0).

f Long chain saturated FA (including C14:0 and longer).

**Table 3:** ISI for the Evaluated Lipid Elements and ASI^a^

	MF1	MF2	MF3	MF (AVE)	VO1	VO2	VO3	VO (AVE)
ISI (FA, g/100 g)	0.98	0.96	0.95	0.96	0.97	0.98	0.98	0.98
ISI (SCFA + MCFA^b^, mg/100 g)	0.83	0.74	0.81	0.79	0.72	0.76	0.53	0.67
ISI (LA, mg/100 g)	0.83	0.77	0.72	0.77	0.84	0.85	0.93	0.87
ISI (ALA, mg/100 g)	0.99	0.97	0.97	0.98	0.87	0.94	0.96	0.92
ISI (ARA, mg/kg)	0.86	0.18	0.32	0.45	0.15	0.81	0.83	0.60
ISI (DHA, mg/kg)	0.96	0.93	0.99	0.96	0.90	0.69	0.87	0.82
ISI (LA/ALA)	0.72	0.65	0.61	0.66	0.63	0.69	0.79	0.70
ISI (total FA composition, %)	0.75	0.71	0.71	0.72	0.51	0.52	0.60	0.54
*ISI(SCSFA* + *MCSFA*^b^*, %)*	*0.80*	*0.76*	*0.86*	*0.81*	*0.68*	*0.74*	*0.53*	*0.65*
*ISI(LCSFA*^c^*, %)*	*0.90*	*0.88*	*0.95*	*0.91*	*0.55*	*0.53*	*0.91*	*0.66*
*ISI(total SFA, %)*	*0.96*	*0.86*	*0.97*	*0.93*	*0.75*	*0.71*	*0.95*	*0.80*
*ISI(total MUFA, %)*	*0.97*	*0.99*	*0.82*	*0.93*	*0.89*	*0.87*	*0.93*	*0.90*
*ISI(n-6 PUFA, %)*	*0.81*	*0.74*	*0.68*	*0.74*	*0.82*	*0.83*	*0.91*	*0.85*
*ISI(n-3 PUFA, %)*	*0.98*	*0.99*	*1.00*	*0.99*	*0.91*	*0.90*	*0.97*	*0.93*
*ISI(total PL content, mg/100 g)*	*0.91*	*0.95*	*0.96*	*0.94*	*0.92*	*0.40*	*0.50*	*0.61*
ISI (PE, mg/100 g)	0.88	0.97	0.88	0.91	0.76	0.20	0.60	0.52
ISI (PC, mg/100 g)	0.75	0.97	0.76	0.83	0.82	0.22	0.58	0.54
ISI (PS mg/100 g)	0.87	0.91	0.99	0.92	0.75	0.51	0.53	0.59
ISI (PI, mg/100 g)	0.64	0.83	0.58	0.68	0.57	0.24	0.66	0.49
ISI (SM, mg/100 g)	0.94	0.74	0.56	0.75	0.64	0.48	0.33	0.48
ISI (GL, mg/100 g)	0.95	0.98	0.86	0.93	0.83	0.44	0.41	0.56
*ISI(PL amount, mg/100 g AVE)*	*0.84*	*0.90*	*0.77*	*0.84*	*0.73*	*0.35*	*0.52*	*0.53*
ISI (PE fatty acids, %)	0.59	0.53	0.54	0.55	0.53	0.54	0.56	0.54
ISI (PC fatty acids, %)	0.61	0.63	0.63	0.63	0.61	0.49	0.57	0.55
ISI (PS fatty acids, %)	0.56	0.52	0.51	0.53	0.54	0.38	0.58	0.50
ISI (PI fatty acids, %)	0.59	0.46	0.39	0.48	0.41	0.77	0.57	0.58
ISI (SM fatty acids, %)	0.47	0.41	0.42	0.43	0.43	0.43	0.50	0.45
ISI (GL fatty acids, %)	0.44	0.43	0.41	0.43	0.40	0.51	0.46	0.46
*ISI(PL fatty acids, % AVE)*	*0.54*	*0.50*	*0.48*	*0.51*	*0.48*	*0.52*	*0.54*	*0.51*
ISI (C10:0 *sn*-2)	0.00	0.00	0.00	0.00	0.00	0.00	0.00	0.00
ISI (C12:0 *sn*-2)	0.43	0.90	0.90	0.75	0.35	0.35	0.24	0.31
ISI (C14:0 *sn*-2)	0.82	0.88	0.97	0.89	0.32	0.25	0.44	0.34
ISI (C16:0 *sn*-2)	0.33	0.36	0.52	0.40	0.05	0.04	0.17	0.09
ISI (C16:1 *sn*-2)	0.67	0.69	0.83	0.73	0.15	0.06	0.08	0.10
ISI (C18:0 *sn*-2)	0.56	0.86	0.26	0.56	0.35	0.34	0.50	0.40
ISI (C18:1 *sn*-2)	0.51	0.43	0.68	0.54	0.35	0.35	0.46	0.39
ISI (C18:2n-6 *sn*-2)	0.40	0.35	0.34	0.36	0.40	0.40	0.44	0.41
ISI (C18:3n-3 *sn*-2)	0.45	0.66	0.51	0.54	0.56	0.48	0.52	0.52
*ISI(sn-2 fatty acids, % AVE)*	*0.46*	*0.57*	*0.56*	*0.53*	*0.28*	*0.25*	*0.32*	*0.28*
*ISI(sn-2 C16:0/tot C16:0, %)*	*0.54*	*0.56*	*0.64*	*0.58*	*0.20*	*0.16*	*0.27*	*0.21*
ISI (C10:0 *sn*-1/3, %)	0.82	0.85	0.68	0.78	0.79	0.86	0.72	0.79
ISI (C12:0 *sn*-1/3, %)	0.94	0.49	0.59	0.67	0.75	0.91	0.65	0.77
ISI (C14:0 *sn*-1/, %)	0.92	0.95	0.96	0.94	1.00	0.91	0.80	0.90
ISI (C16:0 *sn*-1/3, %)	0.81	0.79	0.71	0.77	0.88	0.90	0.68	0.82
ISI (C16:1 *sn*-1/3, %)	0.42	0.42	0.55	0.46	0.23	0.19	0.22	0.21
ISI (C18:0 *sn*-1/3, %)	0.92	0.96	0.85	0.91	0.75	0.83	0.79	0.79
ISI (C18:1 *sn*-1/3, %)	0.91	0.92	0.76	0.86	0.98	0.97	0.77	0.91
ISI (C18:2*n*-6 *sn*-1/3, %)	0.96	0.86	0.82	0.88	1.00	0.98	0.80	0.93
ISI (C18:3*n*-3 *sn*-1/3, %)	0.74	0.94	0.80	0.83	0.66	0.68	0.67	0.67
*ISI(sn-1/3 fatty acids, % AVE)*	*0.83*	*0.80*	*0.75*	*0.79*	*0.78*	*0.80*	*0.68*	*0.75*
ISI (cholesterol, mg/100 g)	0.76	0.64	0.60	0.66	0.34	0.21	0.30	0.28
ISI (droplet size, μm, D[4,3])	0.11	0.15	0.12	0.13	0.16	0.15	0.61	0.31
ASI(fat)	0.69	0.68	0.65	0.68	0.57	0.53	0.57	0.56

a Values marked in italics are excluded from the ASI(fat). Abbreviations: ALA, alpha-linolenic acid; ARA, arachidonic acid; DHA, docosahexaenoic acid; GL, glycolipids; LA, linoleic acid; MF, milk-fat-containing formula; PC, phosphatidyl choline; PE, phosphatidyl ethanolamine; PI, phosphatidyl inositol; PL, polar lipids; PS, phosphatidyl serine; SM, sphingomyelin; and VO, the formula containing vegetable oils as the primary fat source.

b Short chain and medium chain FA (C4:0, C6:0, C8:0, C10:0, and C12:0).

c Long chain saturated FA (including C14:0 and longer).

Nearly 200 different FAs have been detected in breast milk.^25^ However, as the FA composition in breast milk reflects the diet of the mother, there exists no standard value for the FA composition of breast milk. Mature human milk typically contains 34–47% SFA, 31–43% MUFA, 12–26% *n* – 6 PUFA, and 0.8–3.6% *n* – 3 PUFA,^27^ and the proportions vary for example depending on the geographic locations.^28,29^ In our pooled Finnish breast milk, the proportions of SFA, MUFA, *n* – 6 PUFA, and *n* – 3 PUFA were 38.8, 45.7, 12.6, and 2.2%, respectively (Table 2). The MF-containing formulas had a higher similarity index in SFA, MUFA, and *n* – 3 PUFA proportion (0.93, 0.93, and 0.99 on average, respectively) compared to the formulas containing VO as the primary fat source, in which the indexes were 0.80, 0.90, and 0.93 on average, respectively (Table 3). VO-based formulas had a higher similarity index in *n* – 6 PUFA (0.85 on average) compared to MF-containing formulas (0.74 on average). The high content of *n* – 6 PUFA in all of the studied formulas would produce high similarity to the breast milk from, for example, Asia because according to the results of Kumar et al.*,*^29^ Chinese breast milk had significantly higher *n* – 6 PUFA proportion (25.7%) compared to the breast milk from Finland (10.3%), Spain (14.7%), and South Africa (13.4%). A similar trend was found in the other previous studies^28,30^

The highest index for total FA composition, 0.75 of MF1, was obtained by including bovine cream, sunflower oil, milkfat rich whey protein, rapeseed oil, coconut oil, fish oil, and *Mortierella alpina*-oil in the formula (Table 3). The lowest index, 0.50 of VO1, is a result of the mixture of nondairy fats: sunflower oil, rapeseed oil, fish oil, and *M. alpina*-oil. On average, the MF-containing formulas had a similarity index of 0.72 and the VO-based formulas 0.52 for total FA composition.

**Figure 1:**
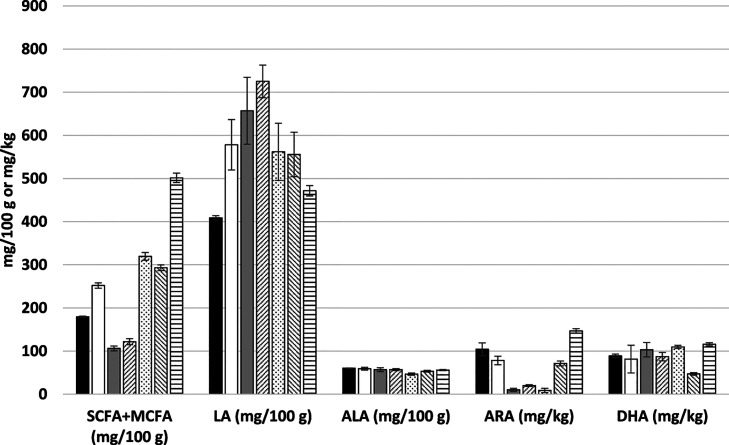
Content of short and medium chain FAs (SCFA + MCFA: C4:0, C6:0, C8:0, C10:0, and C12:0), linoleic acid (LA), arachidonic acid (ARA), and docosahexanoic acid (DHA) in the infant formulas and breast milk. Bar color black, breast milk; white, MF1; grey, MF2; dashed right, MF3; dotted, VO1; dashed left, VO2; and horizontal lines, VO3. Data are average (*n* = 5), and standard deviations are shown. MF, milk-fat-containing formula; VO, the formula containing vegetable oils as the primary fat source.

Figure 1 shows the absolute contents of essential FA: LA and ALA, in the infant formulas and breast milk. The optimal ratio of LA (*n* – 6 FA) to ALA (*n* – 3) FA has been under debate. Namely, there exists evidence that lowering *n* – 6 FA intake in early life could protect from fat mass accumulation in adulthood and increase *n* – 3 FA accumulation in the brain.^31−33^ In Finland, rapeseed oil is the primary VO used in diet and its high ALA content most probably has produced the relatively low (5.5) LA/ALA ratio in the breast milk studied here. In all of the formulas, the LA content was higher than in breast milk (Table 2), but the ALA content was highly similar, which resulted in the higher LA/ALA ratio in the formulas. On average, the ISI (LA/ALA) was higher in the VO-based formulas (0.70) compared to MF-containing formulas (0.66).

In Figure 1, the content of SCFA + MCFA (C4:0 + C6:0 + C8:0 + C10:0 + C12:0), DHA and ARA in the studied infant formulas and breast milk is shown. SCFA + MCFA are absorbed faster than longer chain FAs and have even been speculated to spare ALA from oxidation.^34^ Butyric acid was found exclusively in the formulas containing bovine milk, because it is generated in the rumen biohydrogenation and thus specific to bovine MF^35^ (Table 2). In the infant formulas of this study, the presence of coconut oil increased the MCFA content significantly higher than in breast milk (Figure 1). Therefore, the infant formulas containing MF were higher in similarity to breast milk in SCFA + MCFA (Table 3). The amount of DHA in the formulas was relatively similar (4.7–11.6 mg/100 g) to the breast milk, 8.9 mg/100 g (Figure 1). However, in breast milk, the DHA content is highly dependent on the mother’s recent marine oil consumption and our number cannot be considered a standard value. In none of the formulas the DHA amount was not as high as labeled (14–17 mg/100 g or 100 mL, data not shown). The proposed minimum and maximum values are 12 and 35 mg/100 g (calculated from the given value per 100 kcal) according to EFSA^1^ (2014). The ARA content varied more in the studied milks, but there exists no proposed minimum nor maximum value for ARA.^1^

### Similarity Index for Positional Distribution of FAs

Palmitic acid is the most abundant saturated FA in human milk, and the majority of it, 62–86%, is located in the *sn*-2 position of the TAG molecule.^36−38^ Location of saturated FAs in the middle position of TAG is significant in respect of their optimal adsorption in the infant intestine. Namely, the unesterified long chain SFAs tend to form insoluble salts with Ca2^+^, which, besides limiting adsorption of these nutrients, increases the stool hardness and affects the composition of intestinal microbiota potentially reducing the comfort of infants.^3,39^

**Figure 2:**
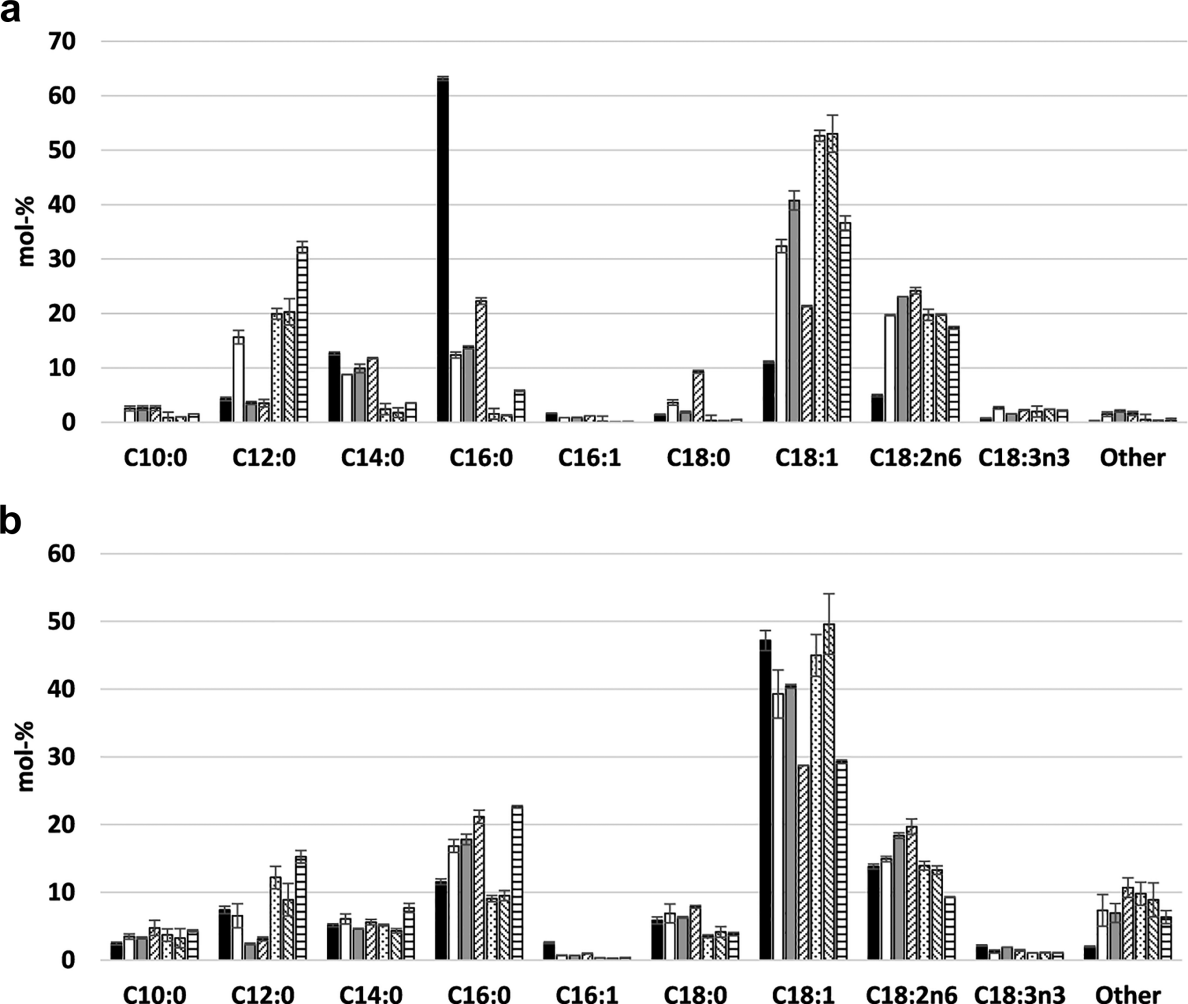
Regioisomerism of triacylglycerols in the infant formulas and breast milk. A. Fatty acids in the *sn*-2 position. B. Fatty acids in the *sn*-1/3 position. Bar color black, breast milk; white, MF1; grey, MF2; dashed right, MF3; dotted, VO1; dashed left, VO2; and horizontal lines, VO3. Data are average (*n* = 3), and standard deviations are shown. MF, milk-fat-containing formula; VO, the formula containing vegetable oils as the primary fat source.

The regiospecific positional distribution of the most significant FAs (>1% in breast milk) of formulas and breast milk in this study are shown in Figure 2A,B. Our study confirmed the location of C16:0 in the *sn*-2 position in breast milk as reported in the literature: 72.6% of C16:0 was found in the *sn*-2 position (Table 2). Of all *sn*-2 FAs, 63.1% was C16:0 in breast milk (Figure 2A, Table 2). Additionally, 71.1% of all C14:0 in breast milk was situated in the *sn*-2 position. Instead, unsaturated FA, such as C18:1 and LA, and MCFA (C10:0 and C12:0) were enriched in the *sn-*1/3 position (Figure 2B).

MF-containing formulas were higher in similarity to breast milk regarding the FA in the *sn*-2 position, especially in respect of C16:0 (Table 3) and C14:0 (data not shown) when compared to VO-based formulas. Still, their *sn*-2 C16:0 content was far from that of breast milk and contained only 26.9% (MF1), 27.9% (MF2), and 34.5% (MF3) of total C16:0 in the *sn*-2 position. Partly resulting from the VO supplementation, the *sn*-2 C18:1 and C18:2 content of also the MF-containing formulas were significantly higher than in breast milk. The VO-based formulas contained only traces of C16:0 in the *sn*-2 position reflecting the typical vegetable fat composition, where the *sn*-2 position is occupied by unsaturated FAs. These formulas also contained less palmitic acid in total (Table 3). In the VO-based formulas, MCFA of which especially C12:0 was enriched in the *sn*-2 position. Supporting optimal absorption in postprandial metabolism, the *sn*-2 position would ideally be occupied by long chain FA, whereas the MCFAs are absorbed at an equal rate despite the positional distribution.^40^ Therefore, the positional distribution of C12:0 in the *sn*-2 position in VOs scarcely brings added value.

The ISI (*sn*-2 Fas, *sn*-1/3 FAs, and *sn*-2 C16:0/total C16:0; %) and their averages are presented in Table 3. It can be concluded that high similarity to human MF is easier to obtain in *sn*-1/3 than *sn*-2 position, and it can be reached without bovine MF supplementation. However, the positioning of palmitic acid in *sn*-2 position, which is critical in infant nutrition, is better, although not optimal, in the bovine MF-containing infant formulas.

### Similarity Index for Polar Lipids and Cholesterol

Polar membrane lipids are very important, yet minor (0.2–1% of total lipids), components in MF, which have several health effects.^41^ In infant formulas, the origin of polar lipids (PL) is typically lecithin derived from the PL fraction of oil plants, which is used as an emulsifier to stabilize VO as small lipid droplets in the formula, and/or MFGM from dairy fat. Despite the primary fat source, all of the formulas in our study except one (VO2) were supplemented with lecithin (Table 1). Table 2 shows the total PL content in the studied infant formulas and breast milk. Our Finnish breast milk contained 56 mg/100 g PL in total. Total PL content in the MF-containing infant formulas studied here was highly similar, ISI (total PL content, mg/100 g) 0.94, on average (Table 3). Lecithin supplementation improved the similarity in total PL content in VO-based formulas, but on average, the similarity index was only 0.61.

**Figure 3:**
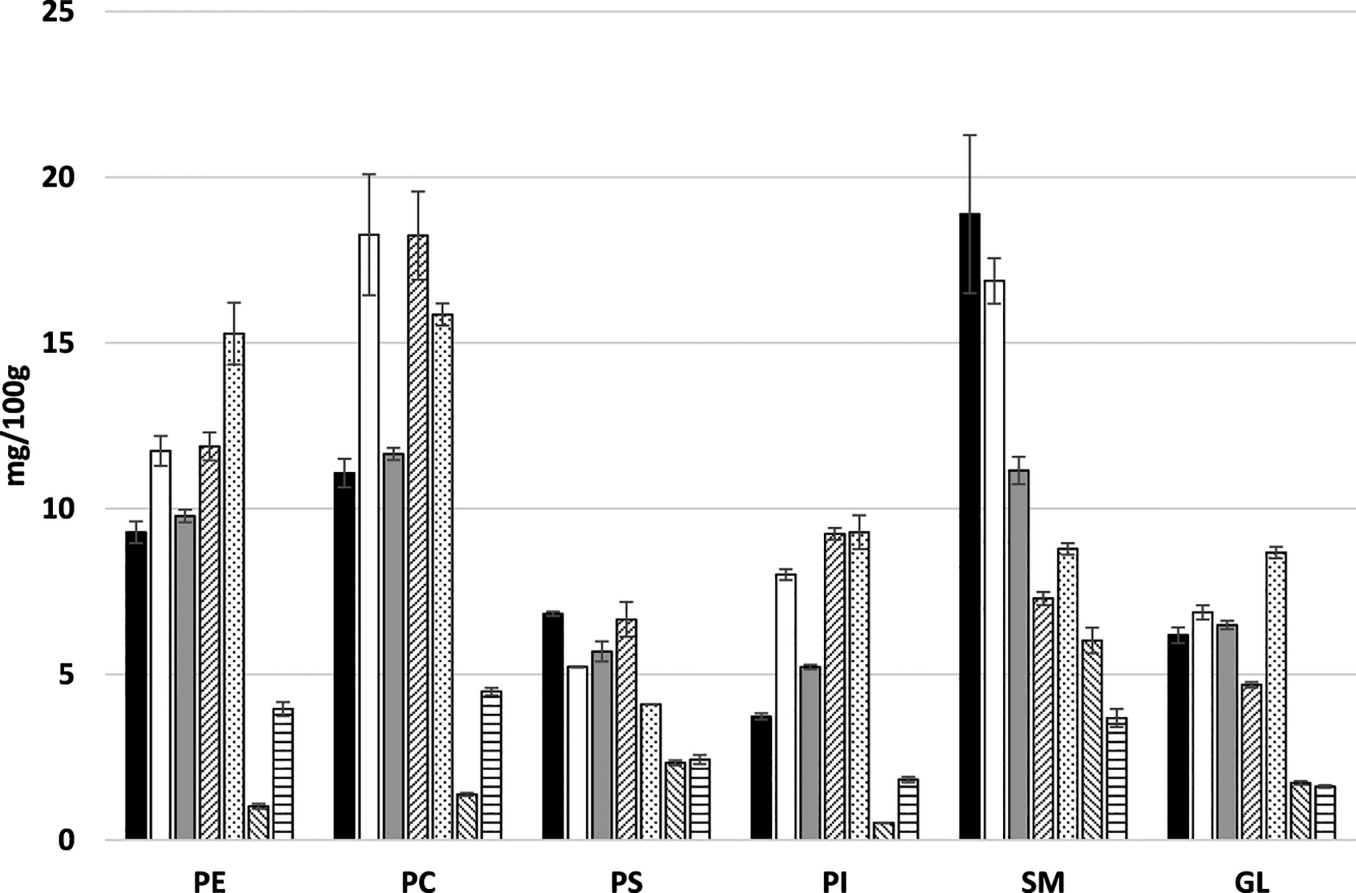
Polar lipids (mg/100 g) in the infant formulas and breast milk. Bar color black, breast milk; white, MF1; grey, MF2; dashed right, MF3; dotted, VO1; dashed left, VO2; and horizontal lines, VO3. Data are average (*n* = 2–4), and standard deviations are shown. Abbreviations: GL, glycolipids, MF, milk-fat-containing formula; PC, phosphatidyl choline; PE, phosphatidyl ethanolamine; PI, phosphatidyl inositol; PS, phosphatidyl serine; SM, sphingomyelin; and VO, the formula containing vegetable oils as the primary fat source.

PL of the MFGM in mammals consists of glycerophospholipids (phosphatidyl choline, phosphatidyl ethanolamine, phosphatidyl serine, and phosphatidyl inositol) and sphingolipids (sphingomyelin and gangliosides), of which the gangliosides are glycosylated.^41^ Lecithin PL composition is different and depends on the botanical source.^42^ In soybean and sunflower lecithin the most abundant PLs are phosphatidyl choline, phosphatidyl inositol, and phosphatidyl ethanolamine representing 90% of PL. According to our results, polar lipid composition was closest to breast milk again in the formulas which contained MF and thus also MFGM (Figure 3, Table 3). ISI (PL amount, mg/100 g AVE) was 0.84, on average, in the MF-containing formulas and 0.53 in the formulas without MF. The clinical studies are usually performed with MFGM extract rather than purified phospholipids. However, the importance of similarity regarding the sphingolipids should be noted because these lipids are evidenced to be important for infants in their cognitive and brain development.^7,43^ These indexes, ISI (SM, mg/100 g) and ISI (GL, mg/100 g) were higher in MF-containing formulas (0.75 and 0.93, respectively, on average) compared to VO-based formulas (0.48 and 0.56, respectively, on average). The reason behind the relatively high sphingomyelin content (6 mg/100 g) in VO1 is not fully clear. We speculate that the whey protein isolate used in the formula might be enriched with MFGM components because this formula had a higher similarity to breast milk also in respect of overall PL composition. We also evaluated the FA composition of each polar lipid (Supporting Information, Figure S1). Also, the polar lipid FA composition was different depending on the polar lipid source, and thus, the differences in ISI (PL FAs, %) were also high: the values varied between 0.38 and 0.77 (Table 3). On average, ISI (PL FAs, % AVE) was the same (0.51) in MF-containing and VO-based formulas.

Besides the similarity index, our data provide interesting information on the FA composition of phospholipids in breast milk. The FA composition was found to be different in bovine MF and human milk-fat-originating sphingomyelin (Supporting Information, Figure S1). As typical, sphingomyelin was rich in long chain saturated FA: C22:0, C23:0, and C24:0. Breast milk sphingomyelin was rich in nervonic acid (C24:1), which has been found to be the most important FA in the brain myelination of the developing human brain.^4,43^ Even if nervonic acid can be synthetized in human metabolism from oleic acid, there is certainly some significance in breast milk containing nervonic acid. Ntoumani et al.^44^ even suggested nervonic acid supplementation in premature infant formulas instead of DHA. In the infant formulas, nervonic acid was also present but only in very small amounts (Supporting Information, Figure S1).

Cholesterol is a structural component associated in cellular membranes with sphingomyelin,^45^ and it is regarded to have health effects, such as short- and long-term reduction of cardiovascular risk factors in infants.^9−11,46^ Instead, the role of vegetable originating phytosterols in infant nutrition is less clear, and there are concerns even of detrimental effects related to their oxidation products.^12^

**Figure 4:**
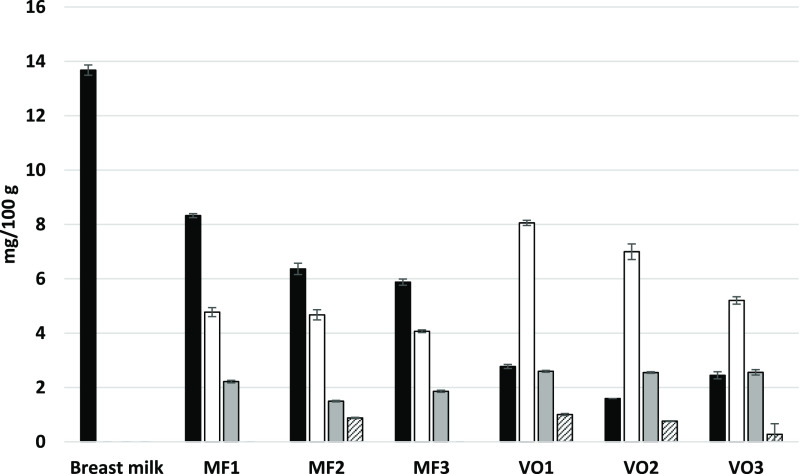
Sterol content in the infant formulas and breast milk (pooled sample of the milk from 8 mothers). Bar color black, cholesterol; white, beta-sitosterol; grey, campesterol; and dashed right, dihydrobrassicasterol. Data are average (*n* = 2). MF, milk-fat-containing formula; VO, the formula containing vegetable oils as the primary fat source.

We analyzed cholesterol and the most abundant phytosterol, beta-sitosterol, content in the infant formulas and breast milk. Also, campesterol and dihydrobrassicasterol could be detected. According to our analysis, breast milk contained a cholesterol level of 13.7 mg/100 g, which is in a range given in the literature, 9.0–22.6 mg/100 g.^47,48^Figure 4 shows that milk-fat-containing infant formulas contained cholesterol, but the amount was significantly lower (8.3, 6.4, and 5.9 for MF1, MF2, and MF3, respectively). In VO-based formulas, the content was still lower (2.8, 1.6, and 2.4 for VO1, VO2, and VO3, respectively). Even if the VO formulas did not contain MF as an ingredient, there were possibly small amounts of MFGM in the fat-free milk and whey, which bring traces of cholesterol in the formulas. Noticeable is the high phytosterol content in all studied formulas. The high content of phytosterols in the formulas may raise a concern about cholesterol adsorption in the infant intestine. Phytosterols are well known for their ability to reduce cholesterol adsorption,^12^ which is beneficial in patients suffering from hypercholesterolemia, but in infant nutrition, this effect can be questionable.

### Lipid Droplet Size

We also measured the lipid droplet size in the formulas and the breast milk. In breast milk, the volume-weighted mean [D4,3] was 5.4 μm. The formulas in liquid form had a droplet size below 0.5 μm (Table 2), which is a prerequisite for the stability of the emulsions during the long shelf life. Therefore, the ISI (droplet size, μm, [D4,3]) was very low (0.11–0.16) in all of the liquid formulas, MF1, MF2, MF3, VO1, and VO2 (Table 3). The powdered formula VO3 had a droplet size of 2.4 μm after it was reconstructed according to the instructions in the package. This was significantly closer to the size of the fat globules in breast milk, and the ISI was 0.61. Powder form enables larger lipid droplet size in respect of storage stability of infant formulas, but the powders may face other stability challenges caused by for example humidity and heat.^49,50^

### Average Similarity Index

After calculating the ISIs for each studied lipid element, the average similarity index, ASI(fat) was calculated for the infant formulas (Table 3). In the calculation of ASI(fat), all the ISIs are averaged in a way that duplication of the values is avoided. for example, the ISIs for the proportions of SFA, MUFA, and PUFA (%) are not calculated for ASI because the same values are already taken into account in the calculation of ISI for total FA composition. The highest ASI(fat) was found in MF1: 0.69. This product contains bovine cream, sunflower oil, MF-rich whey protein, rapeseed oil, coconut oil, fish oil, and *M. alpina*-oil as fat sources, and soy lecithin and mono- and diglycerides as emulsifiers. The ASI(fat) of MF2 and MF3 are following with the ASI(fat) values of 0.68 and 0.65, respectively. Also, these formulas contain bovine MF as a primary fat source. The formulas having VO as the primary fat source have lower ASI(fat) values: 0.57, 0.53, and 0.57, for VO1, VO2, and VO3, respectively. On average, ASI(fat) was 0.68 for MF-containing formulas and 0.56 for VO-based formulas.

In conclusion, this study indicates that having bovine MF as one fat source brings the fat fraction of the infant formulas closer to that of breast milk than the formulas utilizing only VOs. A nutritionally highly important fat fraction in infant formulas may be derived from many different sources in order to fulfill the legislation criteria set for the fat content as well as the content of essential FA and their ratio. By fish or algae oil supplementation, the level of DHA can be raised in order to support the neurodevelopment of the infants. Recognizing the fact that the FA composition of breastmilk has no standard value, the abovementioned fat elements can be adjusted with a high similarity index by using also VOs as the primary fat source. However, when the regiospecific distribution of FA, especially C16:0, and the composition of membrane lipids including cholesterol are evaluated, the MF as an ingredient shows its benefits. Even if these parameters are not controlled by legislation, they play an important role in infant metabolism regarding proper FA adsorption and cellular metabolism through the cell membrane-associated precursors and membrane dynamics. Besides giving information to the manufacturers on how the different fat sources affect the similarity index, this study gives a better understanding of the lipid composition in breast milk. However, there certainly remain several lipid-related components, such as fat-soluble vitamins and other minor lipids, even yet unknown, in breast milk, which are not evaluated here and are important for growing infants. Furthermore, the composition and structural profile of individual molecular species of neutral and polar lipids vary among fat sources, which likely play an important role in infant nutrition. Therefore, breast milk remains the superior option even if the similarity indexes of formulas would be close to unity.

## Supporting Information Available

Fatty acid composition of polar lipids (phosphatidyl choline, phosphatidyl ethanolamine, phosphatidyl inositol, phosphatidyl serine, sphingomyelin, and glycolipids)(PDF)

## Abbreviations Used

ALA: alpha-linolenic acid
ARA: arachidonic acid
ASI: average similarity index
DHA: docosahexaenoic acid
FA: fatty acid
ISI: individual similarity index
LA: linoleic acid
MCFA: medium chain fatty acid
MF: milk fat
MFGM: milk fat globule membrane
MUFA: monounsaturated fatty acid
PL: polar lipid
PUFA: polyunsaturated fatty acid
SCFA: short chain fatty acid
SFA: saturated fatty acid
TAG: triacylglycerol
VO: vegetable oil

## References

[ref1] Scientific Opinion on the essential composition of infant and follow-on formulae. EFSA J. 2014, 12, 24–32. 10.2903/j.efsa.2014.3760.

[ref2] Manson W. G.; Weaver L. T. Fat digestion in the neonate. Arch. Dis. Child. 1997, 76, F206–F211. 10.1136/fn.76.3.f206. PMC1720654 9175955

[ref3] Innis S. M. Dietary triacylglycerol structure and its role in infant nutrition. Adv. Nutr. 2011, 2, 275–283. 10.3945/an.111.000448. 22332059 PMC3090172

[ref4] Martínez M.; Mougan I. Fatty acid composition of human brain phospholipids during normal development. J. Neurochem. 1998, 71, 2528–2533. 10.1046/j.1471-4159.1998.71062528.x. 9832152

[ref5] Youdim K. A.; Martin A.; Joseph J. A. Essential fatty acids and the brain: possible health implications. Int. J. Dev. Neurosci. 2000, 18, 383–399. 10.1016/s0736-5748(00)00013-7. 10817922

[ref6] Fuller K. L.; Kuhlenschmidt T. B.; Kuhlenschmidt M. S.; Jiménez-Flores R.; Donovan S. M. Milk fat globule membrane isolated from buttermilk or whey cream and their lipid components inhibit infectivity of rotavirus in vitro. J. Dairy Sci. 2013, 96, 3488–3497. 10.3168/jds.2012-6122. 23548280

[ref7] Gurnida D. A.; Rowan A. M.; Idjradinata P.; Muchtadi D.; Sekarwana N. Association of complex lipids containing gangliosides with cognitive development of 6-month-old infants. Early Hum. Dev. 2012, 88, 595–601. 10.1016/j.earlhumdev.2012.01.003. 22289412

[ref8] Timby N.; Domellöf E.; Hernell O.; Lönnerdal B.; Domellöf M. Neurodevelopment, nutrition, and growth until 12 mo of age in infants fed a low energy, low-protein formula supplemented with bovine milk fat globule membranes: A randomized controlled trial. Am. J. Clin. Nutr. 2014, 99, 860–868. 10.3945/ajcn.113.064295. 24500150

[ref9] Wong W. W.; Hachey D. L.; Insull W.; Opekun A. R.; Klein P. D. Effect of dietary cholesterol on cholesterol synthesis in breast-fed and formula-fed infants. J. Lipid Res. 1993, 34, 1403–1411. 10.1016/s0022-2275(20)36969-8. 8409771

[ref10] Demmers T. A.; Jones P. J. H.; Wang Y.; Krug S.; Creutzinger V.; Heubi J. E. Effects of early cholesterol intake on cholesterol biosynthesis and plasma lipids among infants until 18 months of age. Pediatrics 2005, 115, 1594–1601. 10.1542/peds.2004-0997. 15930221

[ref11] Owen C. G.; Whincup P. H.; Kaye S. J.; Martin R. M.; Davey Smith G.; Cook D. G.; Bergstrom E.; Black S.; Wadsworth M. E.; Fall C. H.; Freudenheim J. L.; Nie J.; Huxley R. R.; Kolacek S.; Leeson C. P.; Pearce M. S.; Raitakari O. T.; Lisinen I.; Viikari J. S.; Ravelli A. C.; Rudnicka A. R.; Strachan D. P.; Williams S. M. Does initial breastfeeding lead to lower blood cholesterol in adult life? A quantitative review of the evidence. Am. J. Clin. Nutr. 2008, 88, 305–314. 10.1093/ajcn/88.2.305. 18689365

[ref12] Kilvington A.; Maldonado-Pereira L.; Torres-Palacios C.; Medina-Meza I. Phytosterols and their oxidative products in infant formula. J. Food Process Eng. 2020, 43, e13151 10.1111/jfpe.13151.

[ref13] Lopez C.; Briard-Bion V. The composition, supramolecular organisation and thermal properties of milk fat: a new challenge for the quality of food products. Lait 2007, 87, 317–336. 10.1051/lait:2007015.

[ref14] Lopez C.; Cauty C.; Guyomarc’h F. Organization of lipids in milks, infant milk formulas and various dairy products: role of technological processes and potential impacts. Dairy Sci. Technol. 2015, 95, 863–893. 10.1007/s13594-015-0263-0. 26568788 PMC4641158

[ref15] Michalski M. C.; Briard V.; Michel F.; Tasson F.; Poulain P. Size distribution of fat globules in human colostrum, breast milk, and infant formula. J. Dairy Sci. 2005, 88, 1927–1940. 10.3168/jds.s0022-0302(05)72868-x. 15905422

[ref16] Berton A.; Rouvellac S.; Robert B.; Rousseau F.; Lopez C.; Crenon I. Effect of the size and interface composition of milk fat globules on their in vitro digestion by the human pancreatic lipase: native versus homogenized milk fat globules. Food Hydrocolloids 2012, 29, 123–134. 10.1016/j.foodhyd.2012.02.016.

[ref17] Al-Abdi S.; Al-Abdi J.; Al-Aamri M. Similarity Index Between Breast Milk and Infant Formula. EC Paediatrics 2017, 64, 91–111.

[ref18] Kloek W.; Vonk M. M.; Feitsma A. L.; Timmer C. J. A. M. Application of the similarity index to evaluate fat composition and structure in infant formulas. Int. Dairy J. 2020, 111, 104834 10.1016/j.idairyj.2020.104834.

[ref19] Jukkola A.; Hokkanen S.; Kämäräinen T.; Partanen R.; Heino A.; Rojas O. J. Changes in milk fat globules and membrane lipids under the shear fields of microfiltration and centrifugation. J. Membr. Sci. 2019, 573, 218–225. 10.1016/j.memsci.2018.12.007.

[ref20] Suutari M.; Liukkonen K.; Laakso S. Temperature adaptation of yeasts: Role of fatty acids. Microbiology 1990, 136, 1469–1474. 10.1099/00221287-136-8-1469. 2262787

[ref21] Korma S. A.; Zou X.; Ali A. H.; Abed S. M.; Jin Q.; Wang X. Preparation of structured lipids enriched with medium- and long-chain triacylglycerols by enzymatic interesterification for infant formula. Food Bioprod. Process. 2018, 107, 121–130. 10.1016/j.fbp.2017.11.006.

[ref22] Liukkonen K. H.; Montfoort A.; Laakso S. V. Water-Induced Lipid Changes in oat Processing. J. Agric. Food Chem. 1992, 40, 126–130. 10.1021/jf00013a024.

[ref23] Laakso P. Analysis of sterols from various food matrices. Eur. J. Lipid Sci. Technol. 2005, 107, 402–410. 10.1002/ejlt.200501134.

[ref24] Bray J. R.; Curtis J. T. An ordination of upland forest communities of southern Wisconsin. Ecol. Monogr. 1957, 27, 325–349. 10.2307/1942268.

[ref25] Jensen R. G.; Ferris A. M.; Lammi-Keefe C. J.; Henderson R. A. Lipids of bovine and human milks: a comparison. J. Dairy Sci. 1990, 73, 223–240. 10.3168/jds.s0022-0302(90)78666-3. 2184172

[ref26] Damerau A.; Ahonen E.; Kortesniemi M.; Puganen A.; Tarvainen M.; Linderborg K. M. Evaluation of the composition and oxidative status of omega-3 fatty acid supplements on the Finnish market using NMR and SPME-GC–MS in comparison with conventional methods. Food Chem. 2020, 330, 127194 10.1016/j.foodchem.2020.127194. 32544772

[ref27] Delplanque B.; Gibson R.; Koletzko B.; Lapillonne A.; Strandvik B. Lipid Quality in Infant Nutrition: Current Knowledge and Future Opportunities. J. Pediatr. Gastroenterol. Nutr. 2015, 61, 8–17. 10.1097/mpg.0000000000000818. 25883056 PMC4927316

[ref28] Fabritius M.; Linderborg K. M.; Tarvainen M.; Kalpio M.; Zhang Y.; Yang B. Direct inlet negative ion chemical ionization tandem mass spectrometric analysis of triacylglycerol regioisomers in human milk and infant formulas. Food Chem. 2020, 328, 126991 10.1016/j.foodchem.2020.126991. 32512466

[ref29] Kumar H.; du Toit E.; Kulkarni A.; Aakko J.; Linderborg K. M.; Zhang Y.; Nicol M. P.; Isolauri E.; Yang B.; Collado M. C.; Salminen S. Distinct Patterns in Human Milk Microbiota and Fatty Acid Profiles Across Specific Geographic Locations. Front. Microbiol. 2016, 7, 1619 10.3389/fmicb.2016.01619. 27790209 PMC5061857

[ref30] Hageman J. H. J.; Danielsen M.; Nieuwenhuizen A. G.; Feitsma A. L.; Dalsgaard T. K. Comparison of bovine milk fat and vegetable fat for infant formula: Implications for infant health. Int. Dairy J. 2019, 92, 37–49. 10.1016/j.idairyj.2019.01.005.

[ref31] Ailhaud G.; Massiera F.; Weill P.; Legrand P.; Alessandri J.; Guesnet P. Temporal changes in dietary fats: Role of *n*-6 polyunsaturated fatty acids in excessive adipose tissue development and relationship to obesity. Prog. Lipid Res. 2006, 45, 203–236. 10.1016/j.plipres.2006.01.003. 16516300

[ref32] Oosting A.; Kegler D.; van de Heijning B. J. M.; Verkade H. J.; van der Beek E. M. Reduced linoleic acid intake in early postnatal life improves metabolic outcomes in adult rodents following a Western-style diet challenge. Nutr. Res. 2015, 35, 800–811. 10.1016/j.nutres.2015.06.010. 26239950

[ref33] Schipper L.; Oosting A.; Scheurink A. J. W.; van Dijk G.; van der Beek E. M. Reducing dietary intake of linoleic acid of mouse dams during lactation increases offspring brain n-3 LCPUFA content. Prostaglandins, Leukotrienes Essent. Fatty Acids 2016, 110, 8–15. 10.1016/j.plefa.2016.05.001. 27255638

[ref34] Gianni M. L.; Roggero P.; Baudry C.; Fressange-Mazda C.; Galli C.; Agostoni C.; le Ruyet P.; Mosca F. An infant formula containing dairy lipids increased red blood cell membrane omega-3 fatty acids in 4 month-old healthy newborns: A randomized controlled trial. BMC Pediatr. 2018, 18, 53 10.1186/s12887-018-1047-5. 29433457 PMC5810037

[ref35] Lindmark Månsson H. Fatty acids in bovine milk fat. Food Nutr. Res. 2008, 52, 1821 10.3402/fnr.v52i0.1821. PMC2596709 19109654

[ref36] Haddad I.; Mozzon M.; Frega N. G. Trends in fatty acids positional distribution in human colostrum, transitional, and mature milk. Eur. Food Res. Technol. 2012, 235, 325–332. 10.1007/s00217-012-1759-y.

[ref37] Martin J.-C.; Bougnoux P.; Antoine J.-M.; Lanson M.; Couet C. Triacylglycerol structure of human colostrum and mature milk. Lipids 1993, 28, 637–643. 10.1007/bf02536059. 8355593

[ref38] Wu K.; Gao R.; Tian F.; Mao Y.; Wang B.; Zhou L.; Shen L.; Guan Y.; Cai M. Fatty acid positional distribution (*sn*-2 fatty acids) and phospholipid composition in Chinese breast milk from colostrum to mature stage. Br. J. Nutr. 2019, 121, 65–73. 10.1017/s0007114518002994. 30378505

[ref39] Bar-Yoseph F.; Lifshitz Y.; Cohen T. Review of sn-2 palmitate oil implications for infant health. Prostaglandins, Leukotrienes Essent. Fatty Acids 2013, 89, 139–143. 10.1016/j.plefa.2013.03.002. 23541258

[ref40] Linderborg K. M.; Kallio H. P. T. Triacylglycerol Fatty Acid Positional Distribution and Postprandial Lipid Metabolism. Food Rev. Int. 2005, 21, 331–355. 10.1080/fri-200061623.

[ref41] Brink L. R.; Lönnerdal B. Milk fat globule membrane: the role of its various components in infant health and development. J. Nutr. Biochem. 2020, 85, 108465 10.1016/j.jnutbio.2020.108465. 32758540

[ref42] Bot F.; Cossuta D.; O’Mahony J. A. Inter-relationships between composition, physicochemical properties and functionality of lecithin ingredients. Trends Food Sci. Technol. 2021, 111, 261–270. 10.1016/j.tifs.2021.02.028.

[ref43] Schneider N.; Hauser J.; Oliveira M.; Cazaubon E.; Mottaz S. C.; O’Neill B. V.; Steiner P.; Deoni S. C. L. Sphingomyelin in Brain and Cognitive Development: Preliminary Data. eNeuro 2019, 6, 0421–518. 10.1523/eneuro.0421-18.2019. PMC6709232 31324675

[ref44] Ntoumani E.; Strandvik B.; Sabel K.-G. Nervonic acid is much lower in donor milk than in milk from mothers delivering premature infants -of neglected importance?. Prostaglandins, Leukotrienes Essent. Fatty Acids 2013, 89, 241–244. 10.1016/j.plefa.2013.06.005. 23870193

[ref45] Lopez C.; Madec M.-N.; Jimenez-Flores R. Lipid rafts in the bovine milk fat globule membrane revealed by the lateral segregation of phospholipids and heterogeneous distribution of glycoproteins. Food Chem. 2010, 120, 22–33. 10.1016/j.foodchem.2009.09.065.

[ref46] Wong W. W.; Hachey D. L.; Insull W.; Opekun A. R.; Klein P. D. Effect of dietary cholesterol on cholesterol synthesis in breast-fed and formula-fed infants. J. Lipid Res. 1993, 34, 1403–1411. 10.1016/s0022-2275(20)36969-8. 8409771

[ref47] Koletzko B. Human milk lipids. Ann. Nutr. Metab. 2016, 69, 28–40. 10.1159/000452819. 28103608

[ref48] Zhang N.; Zhuo C. F.; Liu B.; Ye W. H.; Tao L.; Zheng L. F.; Chen L.; Deng Z. Y.; Li G. Y.; Gong Z. Q.; Li J. Temporal Changes of Phospholipids Fatty Acids and Cholesterol in Breast Milk and Relationship with Diet. Eur. J. Lipid Sci. Technol. 2020, 122, 1900187 10.1002/ejlt.201900187.

[ref49] Nugroho R. W. N.; Outinen M.; Toikkanen O.; Heino A.; Sawada D.; Rojas O. J. Effect of water activity on the functional, colloidal, physical, and microstructural properties of infant formula powder. J. Colloid Interface Sci. 2021, 586, 56–66. 10.1016/j.jcis.2020.10.069. 33143850

[ref50] Phosanam A.; Chandrapala J.; Huppertz T.; Adhikari B.; Zisu B. Changes in Physicochemical and Surface Characteristics in Model Infant Milk Formula Powder (IMF) During Storage. Dry. Technol. 2020, 39, 2119–2129. 10.1080/07373937.2020.1755978.

