# Supplementary material for: Similarity Index for the Fat Fraction between Breast Milk and Infant Formulas
Source: J Agric Food Chem. 2022 May 11;70(20):6191–201. doi: 10.1021/acs.jafc.1c08029 (PMC9136929; doi:10.1021/acs.jafc.1c08029)
Supplement: Supplementary file 1 — jf1c08029_si_001.pdf [file jf1c08029_si_001.pdf]

SUPPLEMENTARY DATA

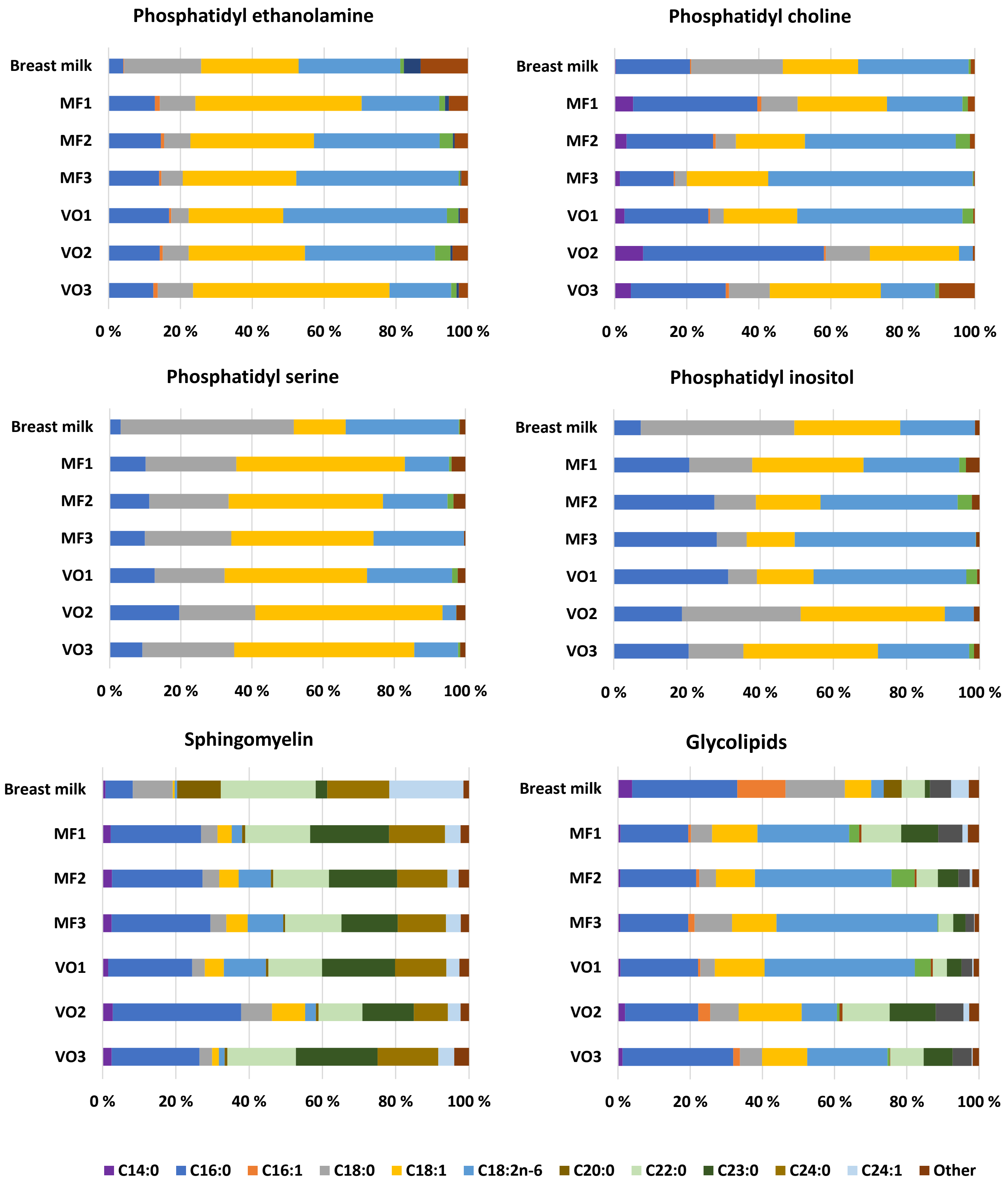

Figure S1. Fatty acid composition of the polar lipids in the infant formulas and breast milk. Data are average (n=2-4). MF, milk fat containing formula; VO, Formula containing vegetable oils as primary fat source.
